# Supplementary material for: Understanding the genetic sex-determining mechanism in Hyla eximia treefrog inferred from H-Y antigen
Source: PLoS One. 2024 May 31;19(5):e0304554. doi: 10.1371/journal.pone.0304554 (PMC11142436; doi:10.1371/journal.pone.0304554)
Supplement: S1 Raw images — (PDF) [file pone.0304554.s001.pdf]

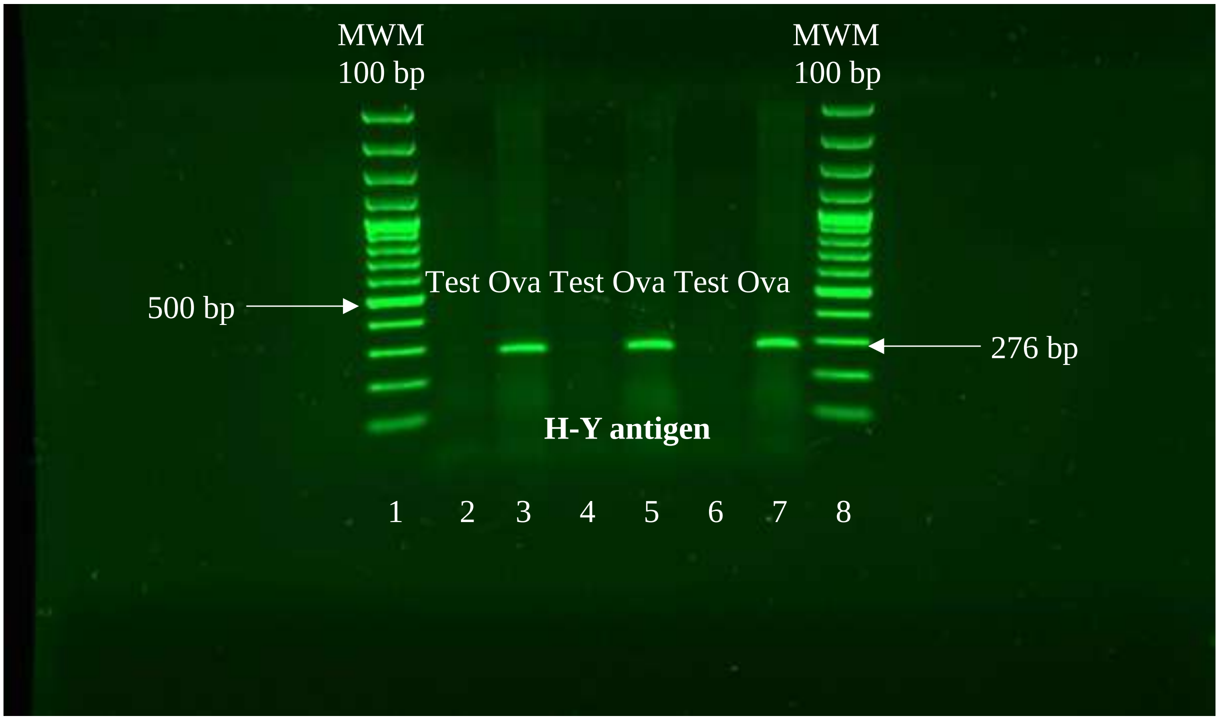

The PCR reactions were visually assessed on 1% agarose gels containing 0.5 µL/100 mL ethidium bromide run at 100 V for 1 hour. The PCR amplifications were observed using a UV transilluminator (Molecular Imager Gel Doc XR System, BioRad Laboratories, Hercules, CA, USA). Method used to capture the image: 1) Image lab software, 2) new protocol, 3) select nucleic acid, 4) ethidium bromide, 5) position gel, and 6) run protocol. Amplicon size was determined by comparison to a 100 base pairs molecular weight marker.

Fig. 2D
