# Supplementary material for: Understanding the genetic sex-determining mechanism in Hyla eximia treefrog inferred from H-Y antigen
Source: PLoS One. 2024 May 31;19(5):e0304554. doi: 10.1371/journal.pone.0304554 (PMC11142436; doi:10.1371/journal.pone.0304554)
Supplement: S1 File — (DOCX) [file pone.0304554.s002.docx]

| 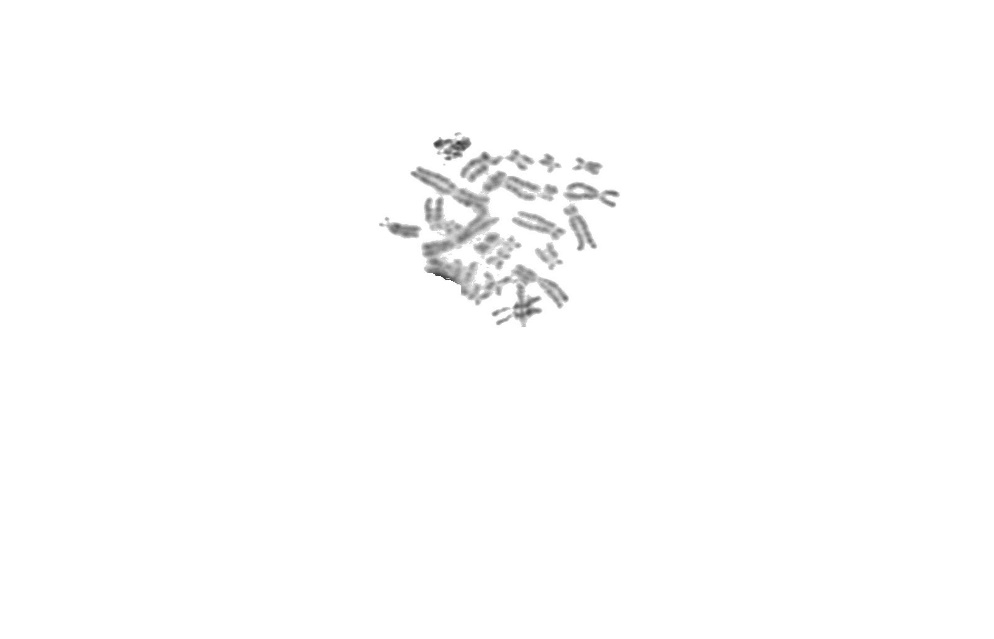 | 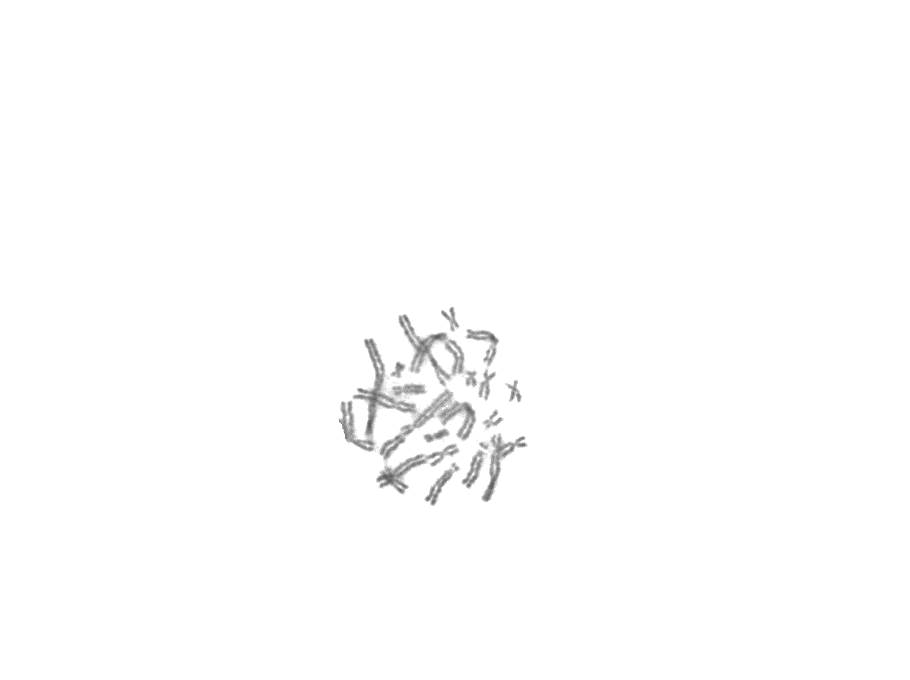 | 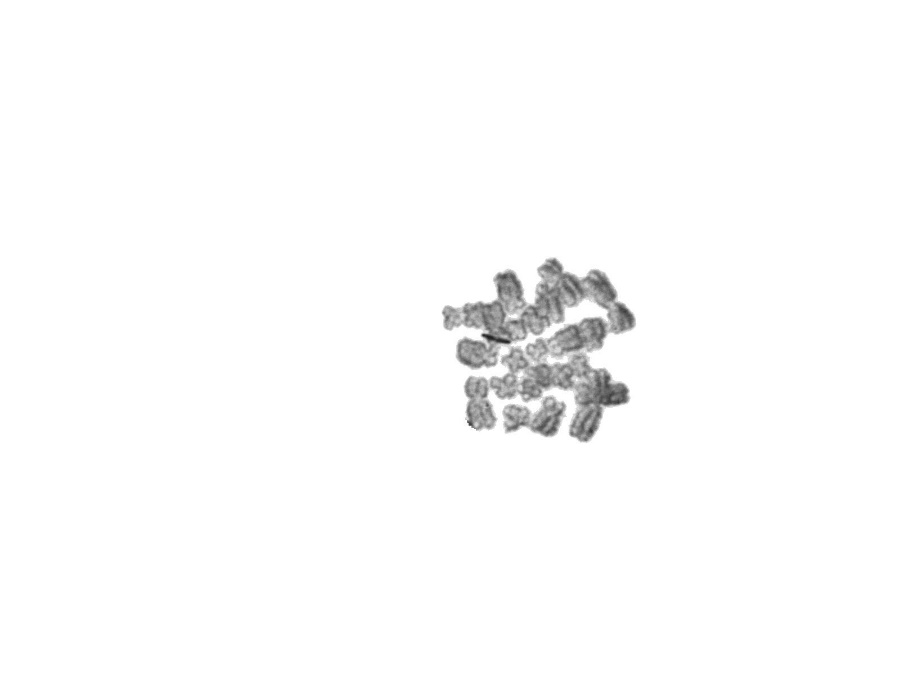 |
| --- | --- | --- |
| 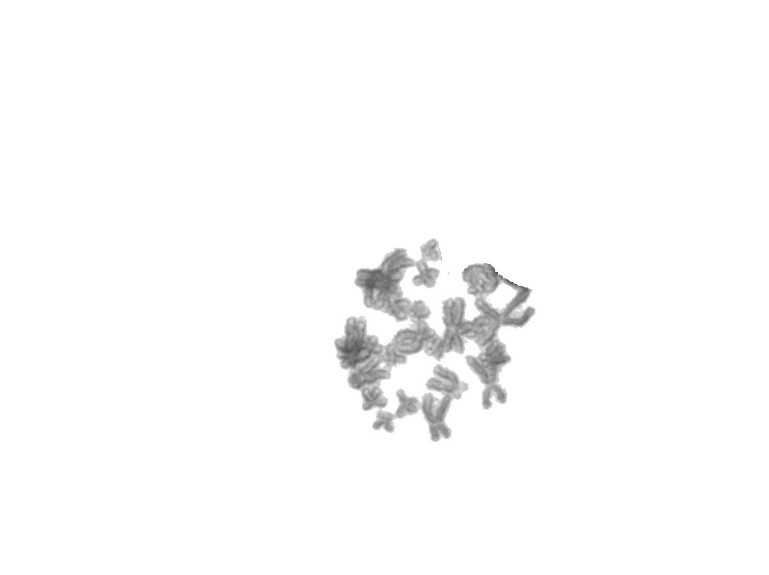 | 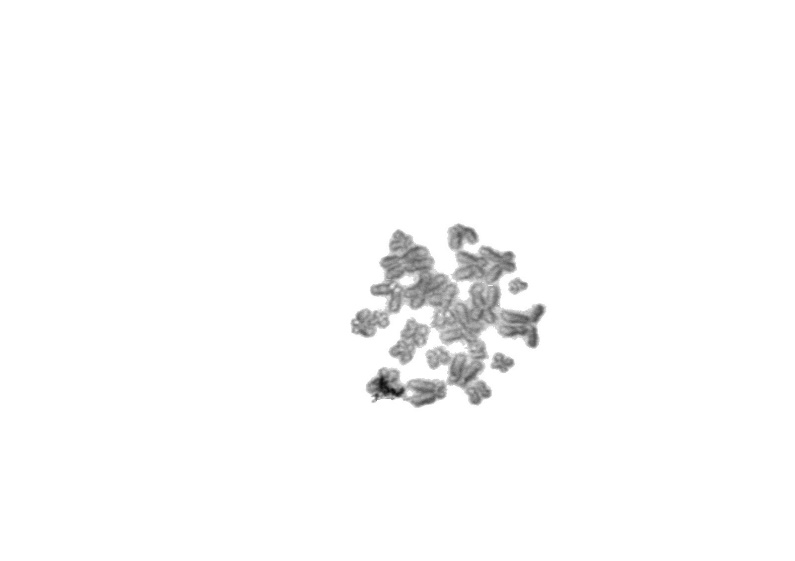 | 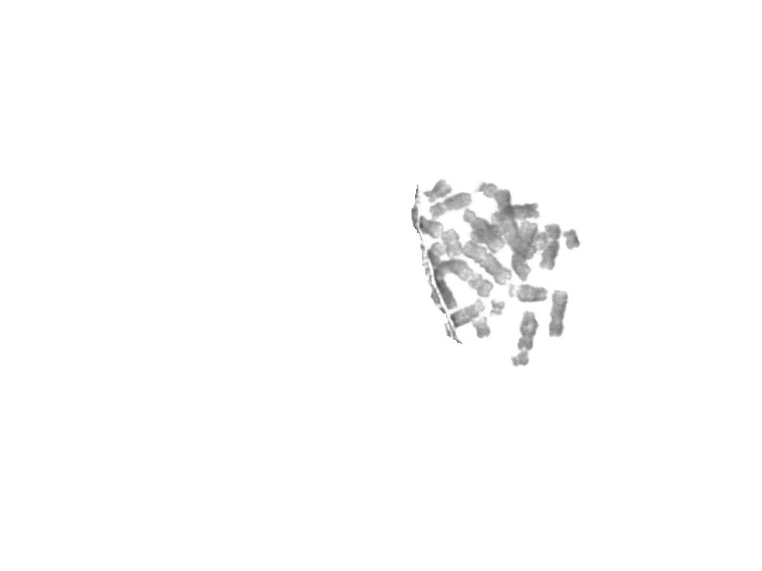 |
| 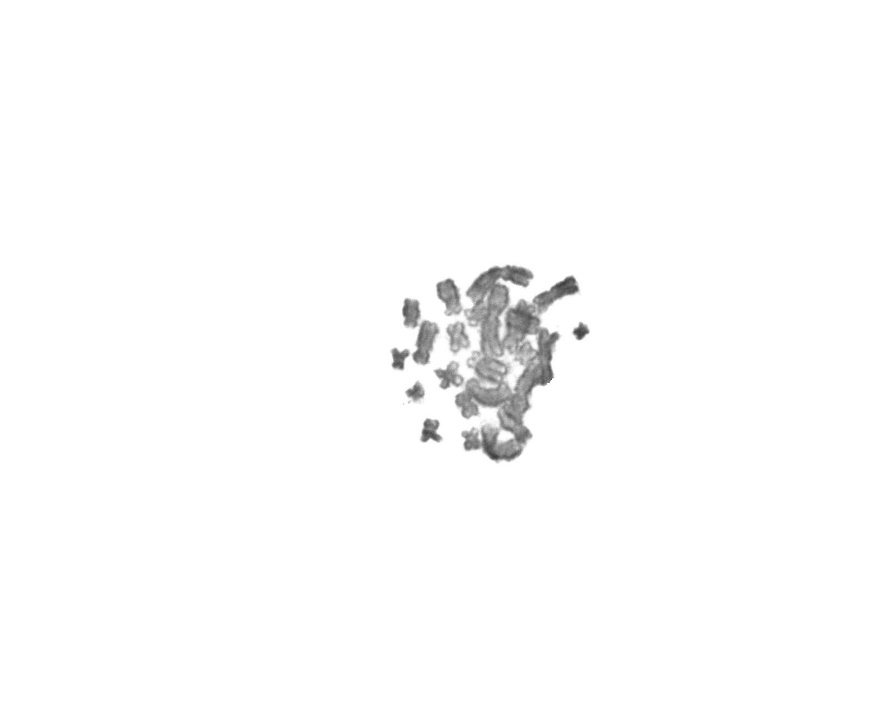 | 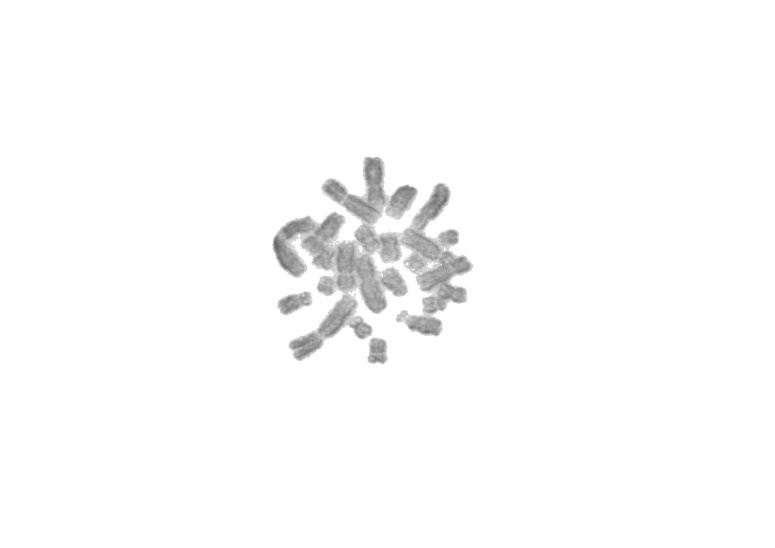 | 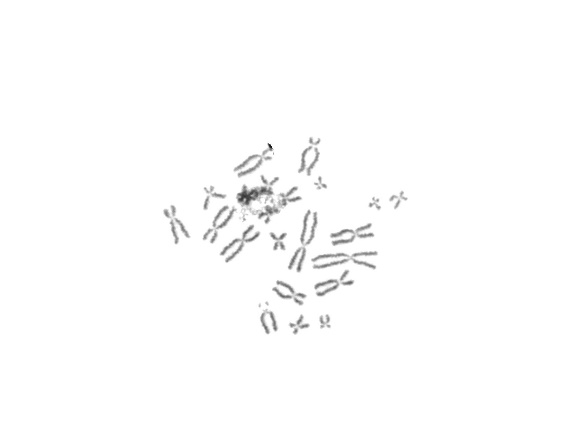 |
| 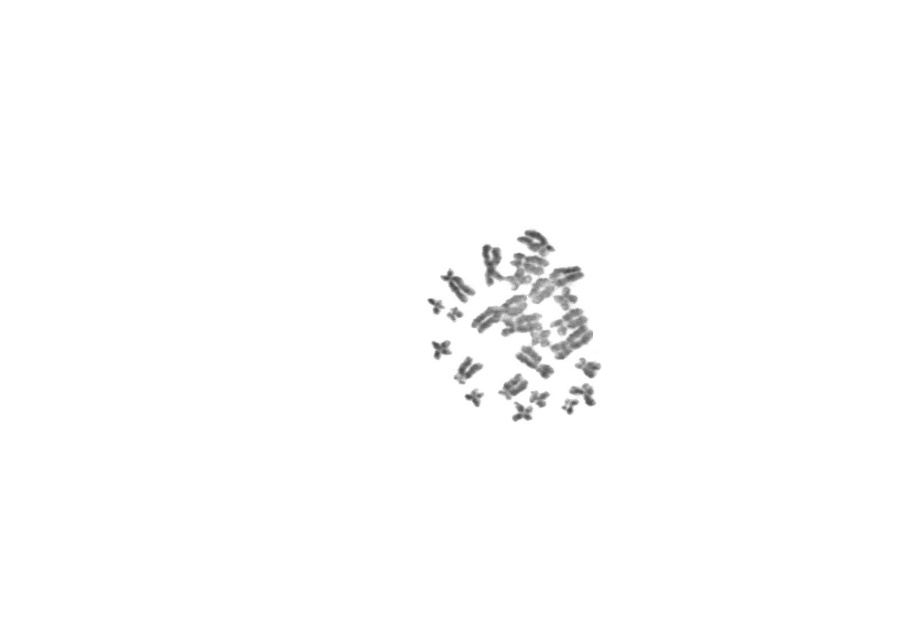 | 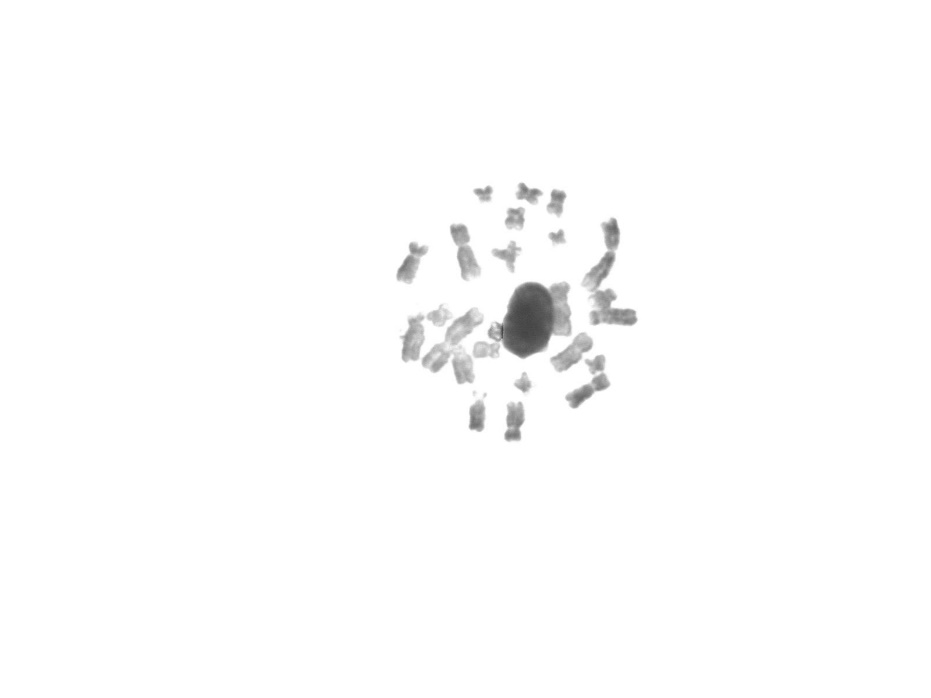 | 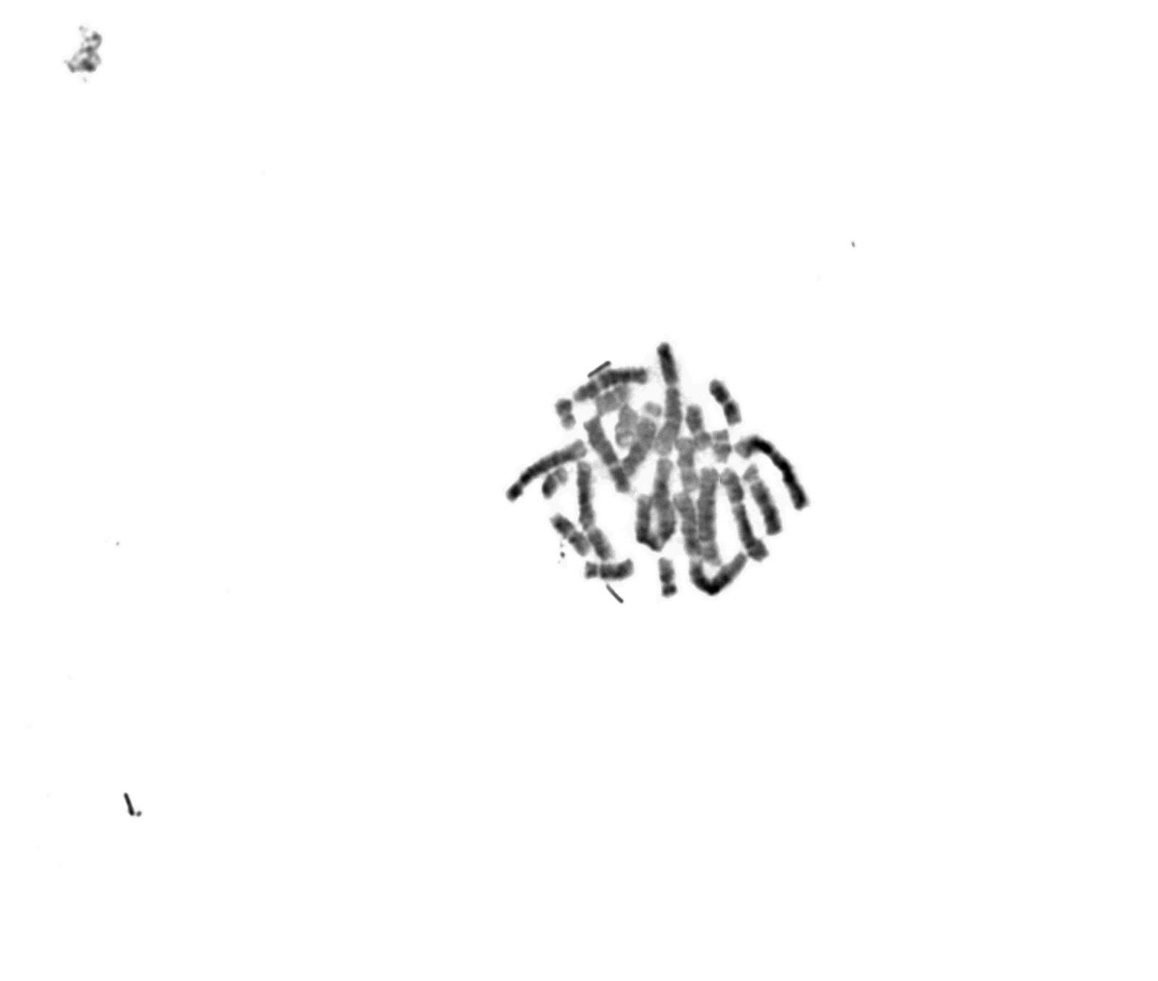 |
| 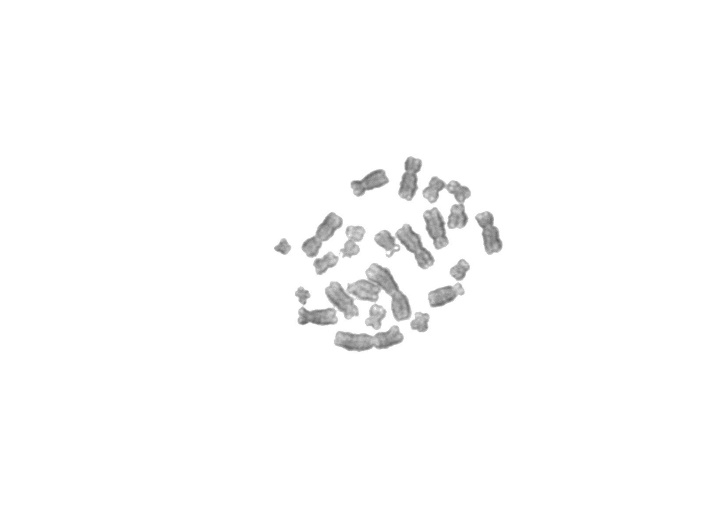 | 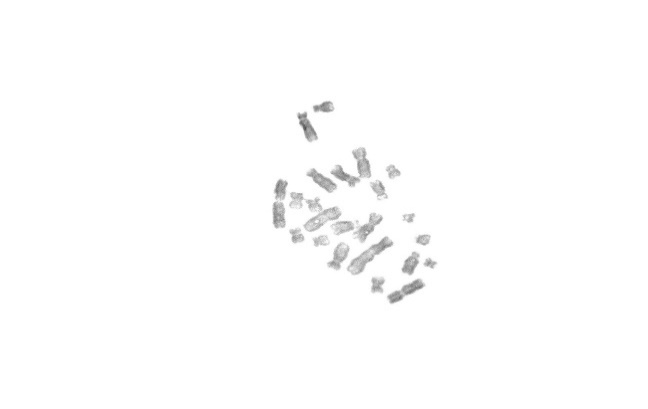 | 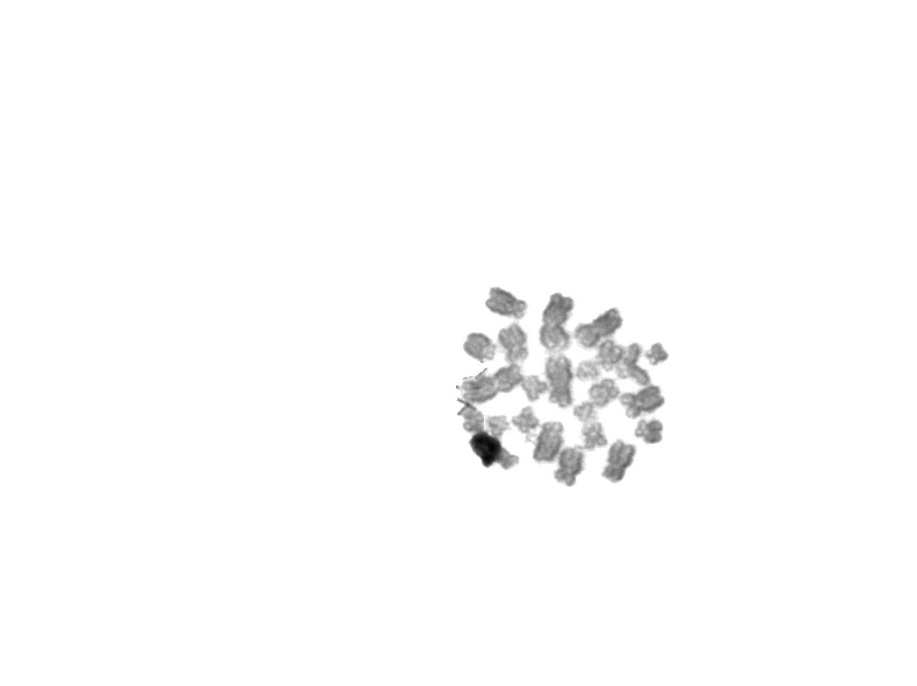 |

**Supplementary figure 1.** Collection of metaphase chromosomes from a male adult frog, stained with Giemsa and visualized under an optical microscope using the 100X immersion objective. Fifteen images of metaphase chromosomes are displayed.

| 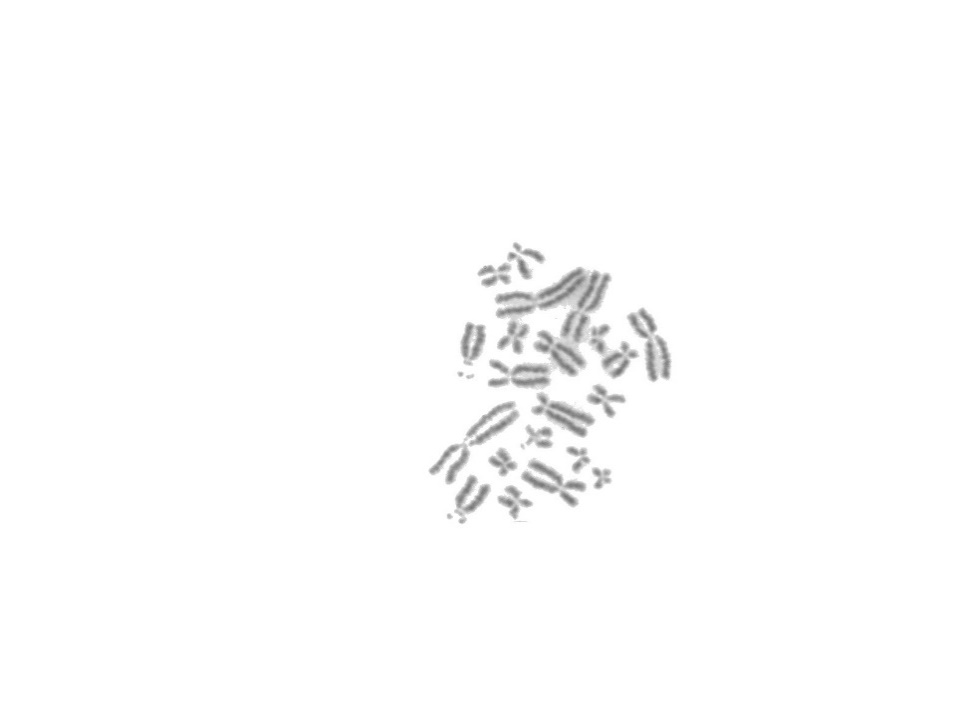 | 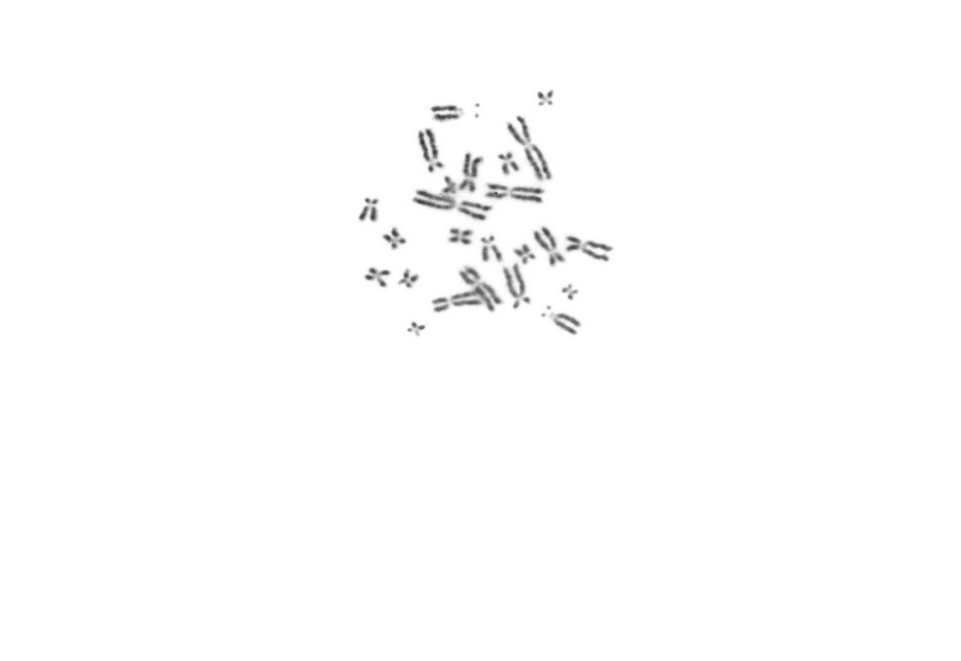 | 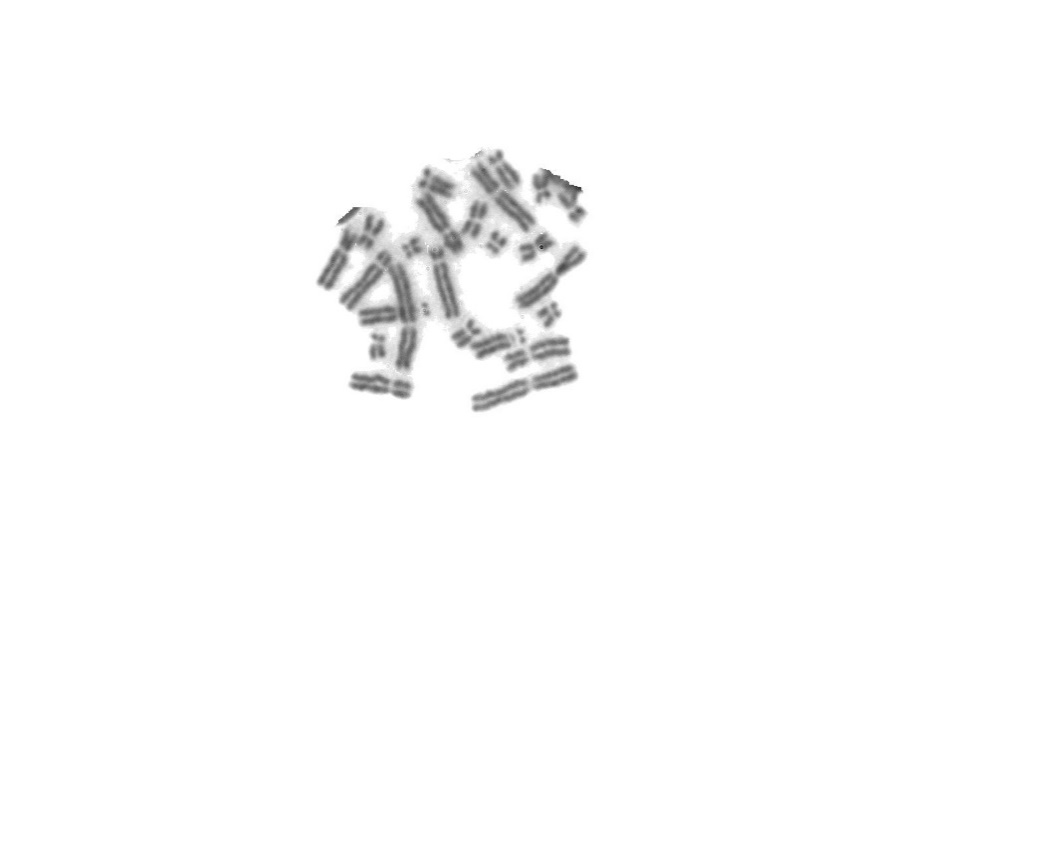 |
| --- | --- | --- |
| 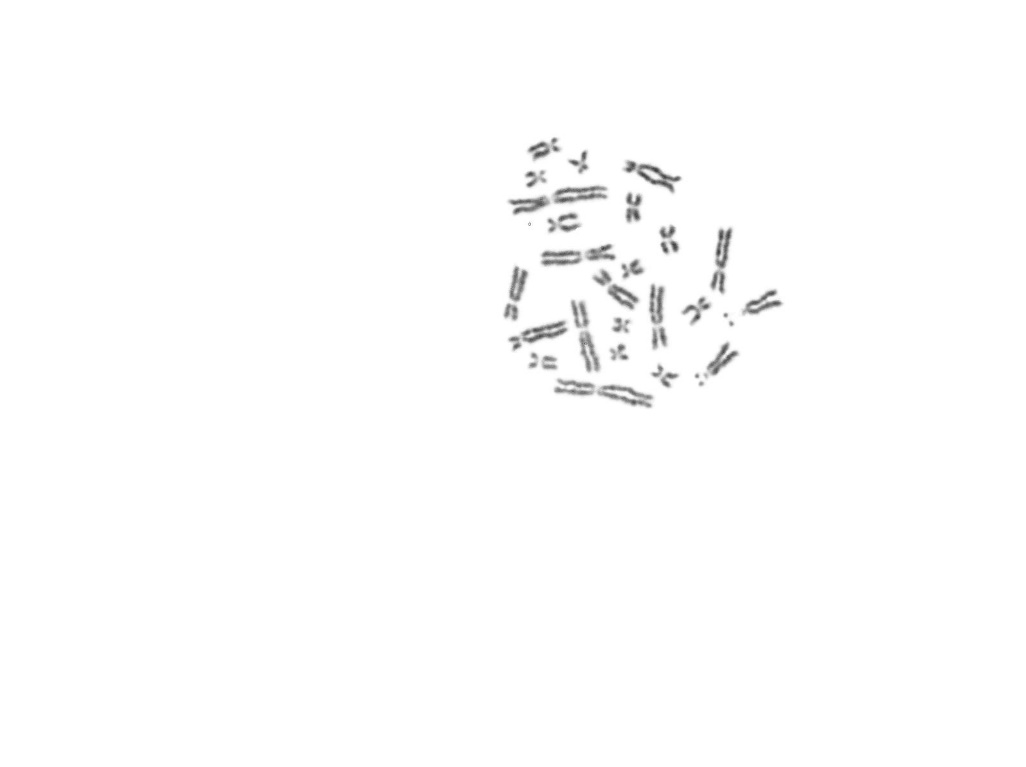 | 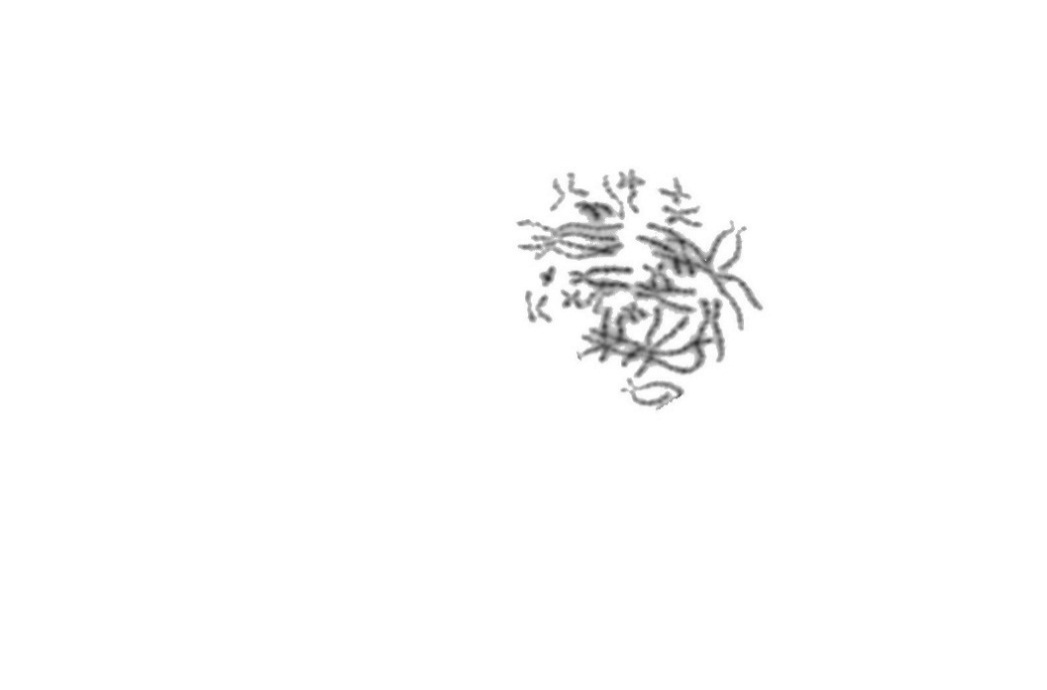 | 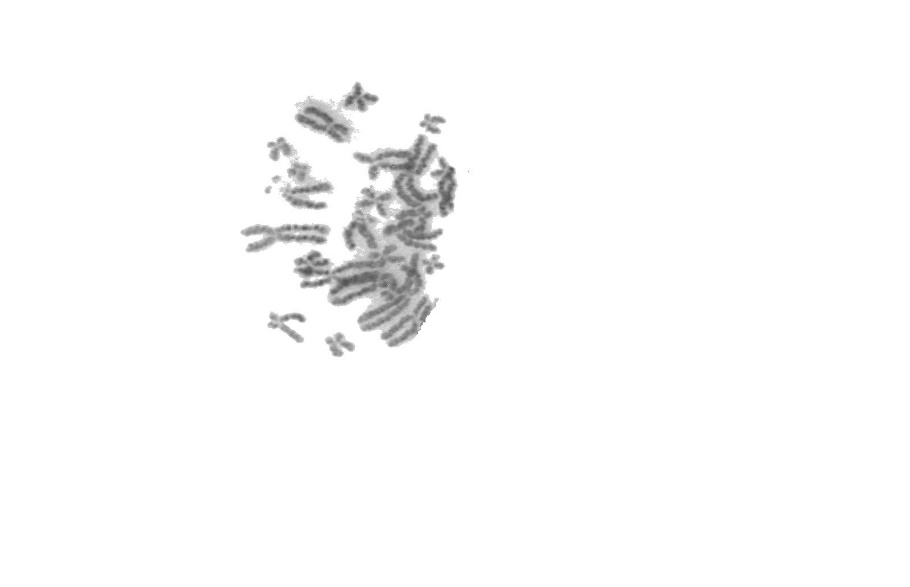 |
| 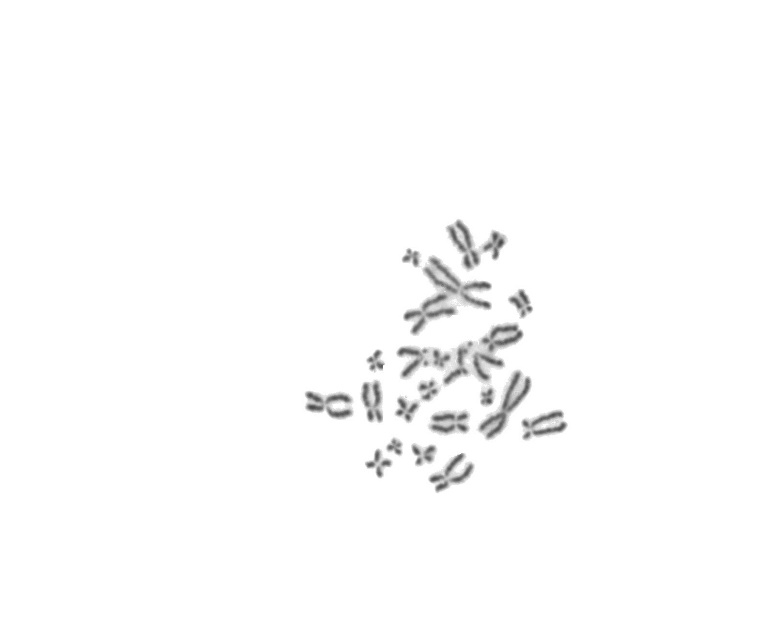 | 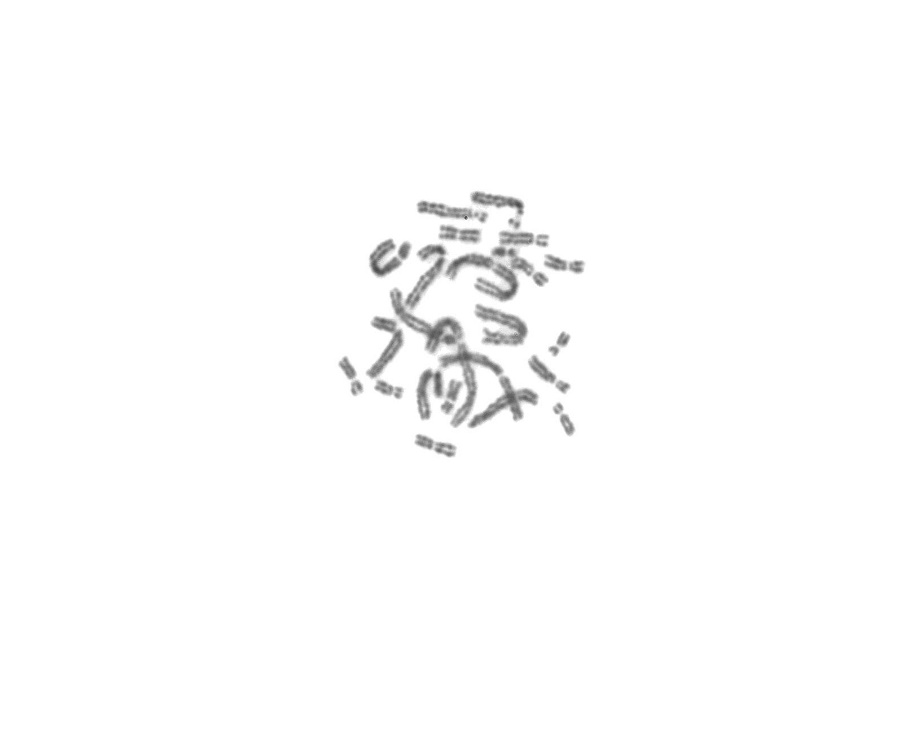 | 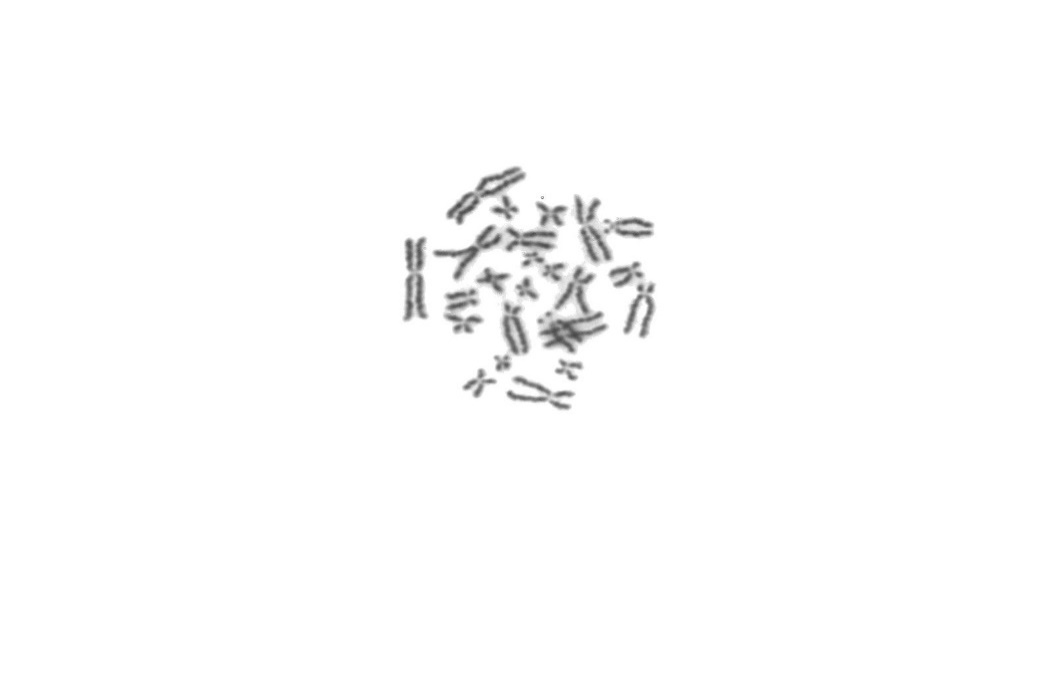 |
| 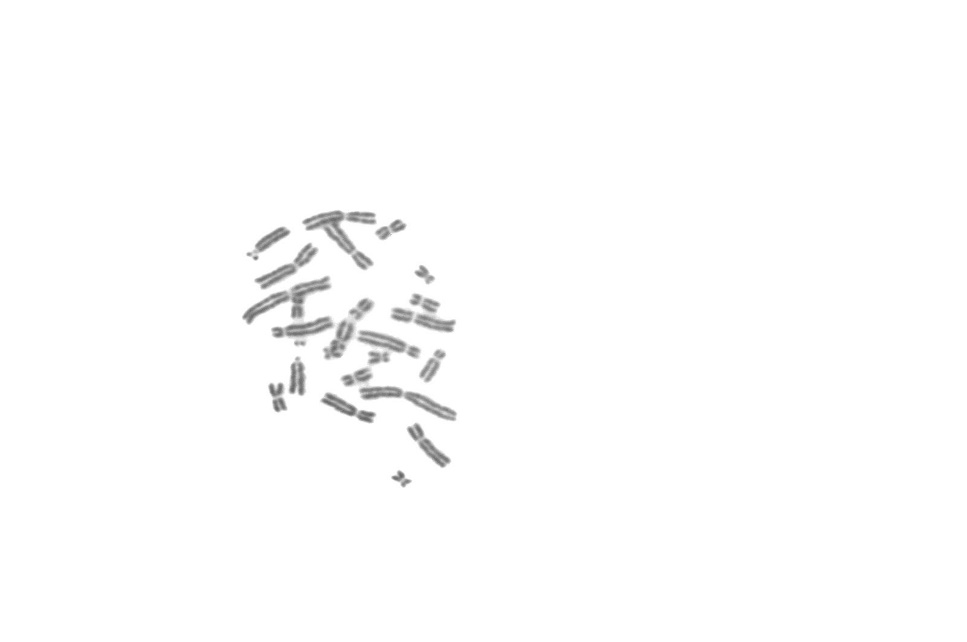 | 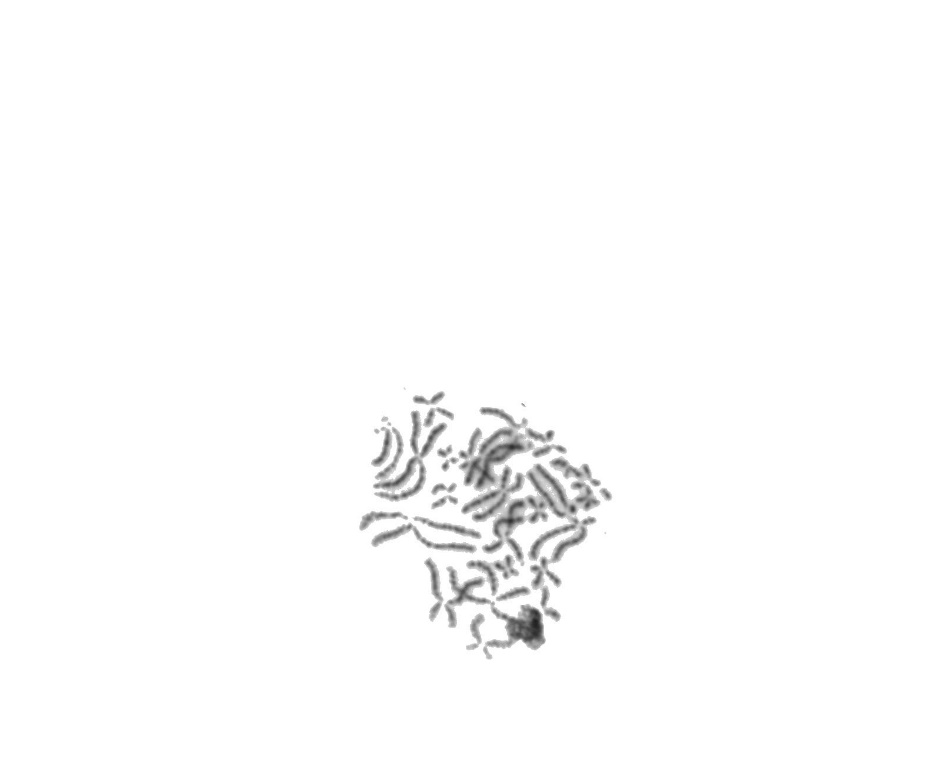 | 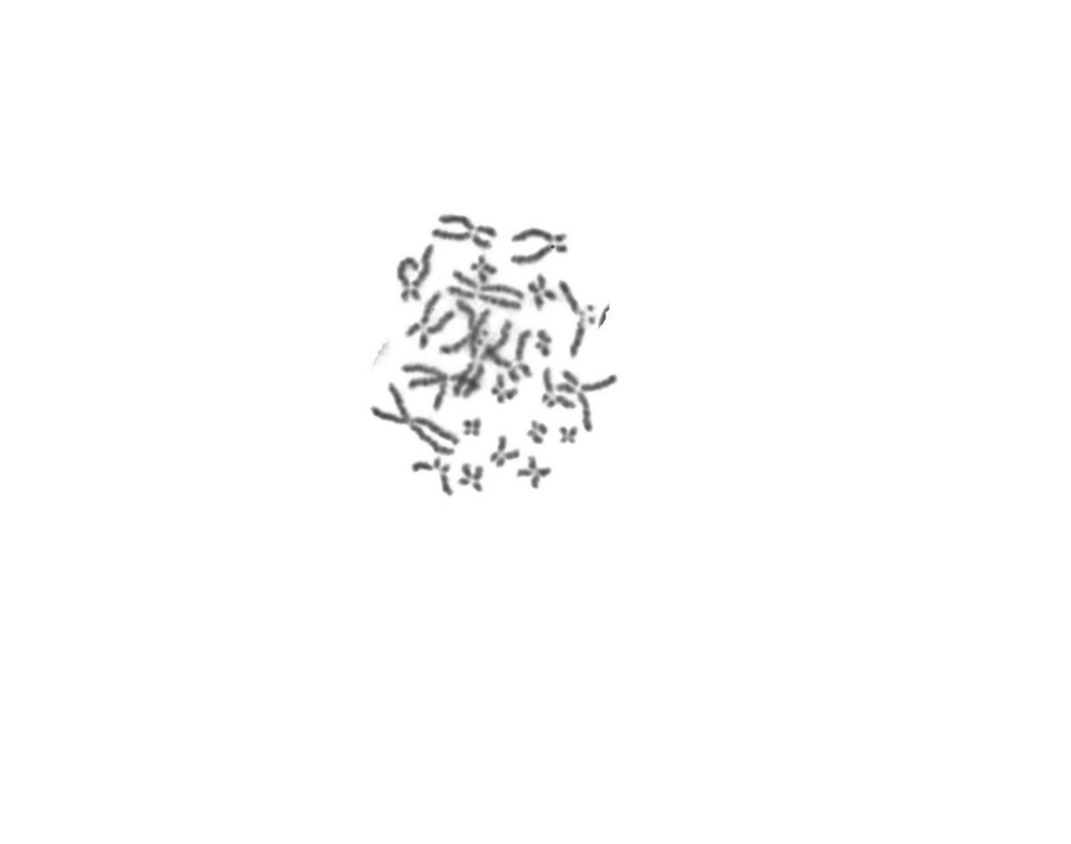 |
| 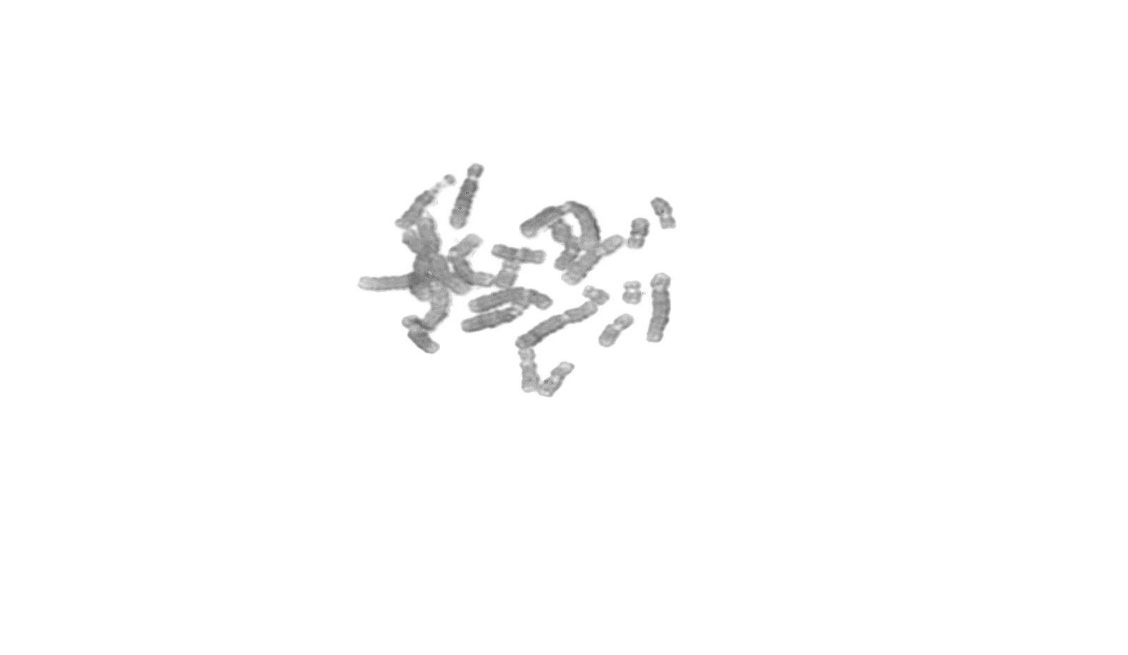 | 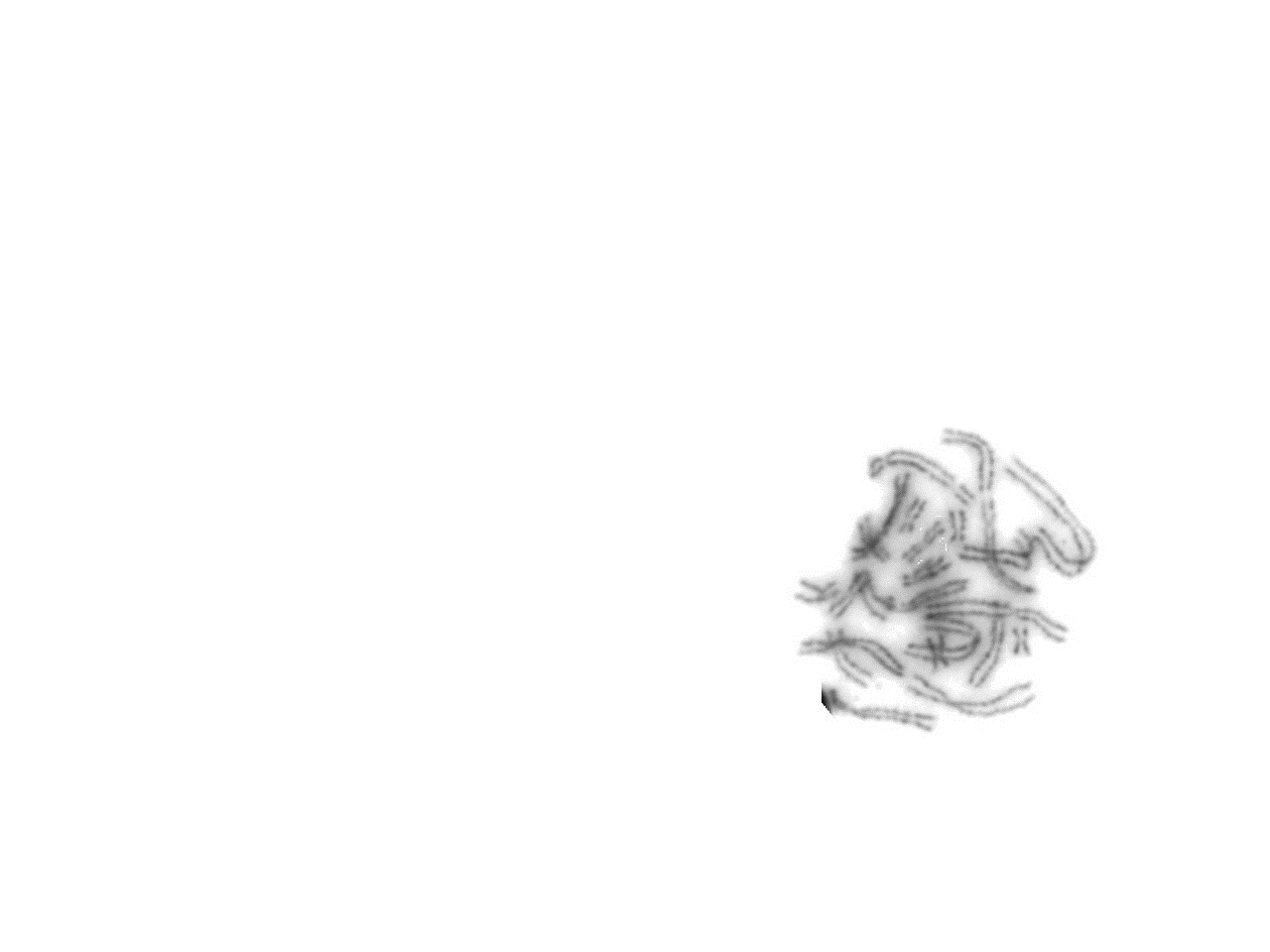 | 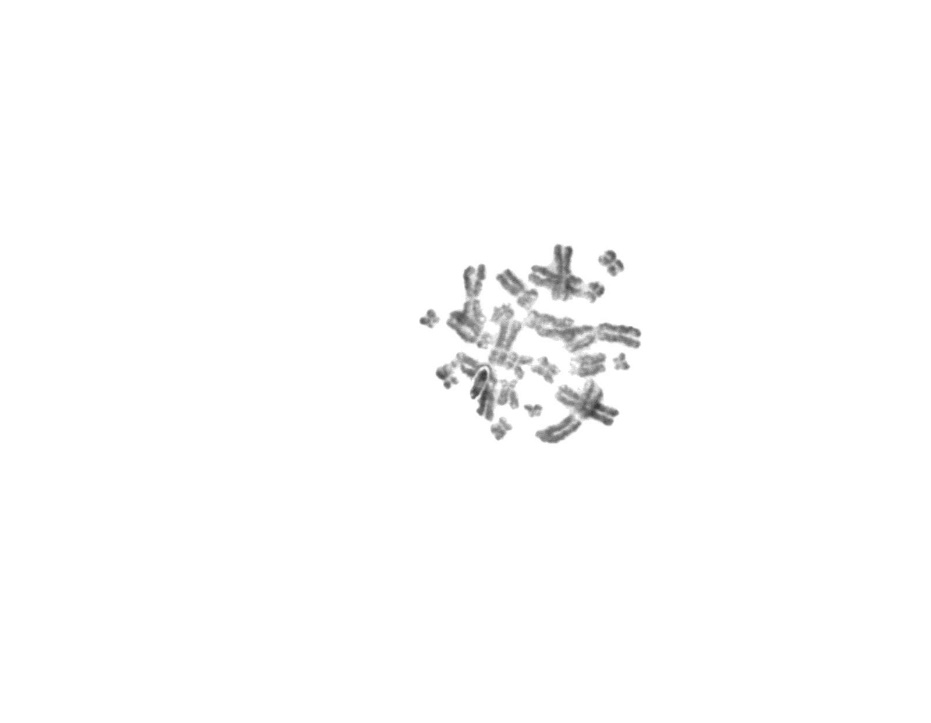 |

**Supplementary figure 2.** Collection of metaphase chromosomes from a female adult frog, stained with Giemsa and visualized under an optical microscope using the 100X immersion objective. Fifteen images of metaphase chromosomes are displayed.
