# Supplementary material for: Understanding the genetic sex-determining mechanism in Hyla eximia treefrog inferred from H-Y antigen
Source: PLoS One. 2024 May 31;19(5):e0304554. doi: 10.1371/journal.pone.0304554 (PMC11142436; doi:10.1371/journal.pone.0304554)
Supplement: Attachment — Submitted filename: Response to Reviewers.docx [file pone.0304554.s003.docx]

**Response to Reviewer #2 and Reviewer #3:**

We greatly appreciate the comments from the reviewers. Likewise, we have kindly considered all the comments to the manuscript and have modified the paper in detail based on all suggestions. We believe the manuscript is now much improved. All changes to the revised paper are indicated by pink text.
